# Desarda Versus Lichtenstein Technique for Primary Inguinal Hernia Treatment: 3-Year Results of a Randomized Clinical Trial

**DOI:** 10.1007/s00268-012-1508-1

**Published:** 2012-03-03

**Authors:** Jacek Szopinski, Stanislaw Dabrowiecki, Stanislaw Pierscinski, Marek Jackowski, Maciej Jaworski, Zbigniew Szuflet

**Affiliations:** 1 Department of General and Endocrine Surgery, Ludwik Rydygier Collegium Medicum in Bydgoszcz, Nicolaus Copernicus University of Torun, ul. M. Sklodowskiej-Curie 9, 85-094 Bydgoszcz, Poland; 2 Department of General, Gastrointestinal, and Cancer Surgery, Ludwik Rydygier Collegium Medicum in Bydgoszcz, Nicolaus Copernicus University of Torun, ul. Sw. Jozefa 53/59, 87-100 Torun, Poland; 3 Department of General Surgery, Jonscher Community Hospital, ul. Milionowa 14, 93-113 Lodz, Poland

## Abstract

**Background:**

The Shouldice method and other tissue-based techniques are still acknowledged to be acceptable for primary inguinal hernia repair according to the European Hernia Society guidelines. Desarda’s technique, presented in 2001, is an original hernia repair method using an undetached strip of external oblique aponeurosis. This randomized trial compared outcomes after hernia repair with Desarda (D) and mesh-based Lichtenstein (L) techniques.

**Methods:**

A total of 208 male patients were randomly assigned to the D or L group (105 vs. 103, respectively). The primary outcomes measured were recurrence and chronic pain. Additionally, early and late complications, foreign body sensation, and return to everyday activity were examined in hospital and at 7, 30 days, and 6, 12, 24, and 36 months after surgery.

**Results:**

During the follow-up, two recurrences were observed in each group (*p* = 1.000). Chronic pain was experienced by 4.8 and 2.9% of patients from groups D and L, respectively (*p* = 0.464). Foreign body sensation and return to activity were not different between the groups. There was significantly less seroma production in the D group (*p* = 0.004).

**Conclusions:**

The results of primary inguinal hernia repair with the Desarda and Lichtenstein techniques are comparable at the 3-year follow-up. The technique may potentially increase the number of tissue-based methods available for treating groin hernias.

## Introduction

Because of their frequency, inguinal hernias remain an important medical problem. The estimated lifetime risk for inguinal hernia is 27% for men and 3% for women [1]. Annual morbidity rates in various countries vary from 100 to 300 per 100,000 citizens [2]. There were no written surgical guidelines for hernia treatment until 2009, when the European Hernia Society (EHS) published its recommendations based on analysis of the literature and the results of clinical trials. In the EHS guidelines, mesh-based techniques—the Lichtenstein technique in particular—and endoscopic methods are recommended for treatment of symptomatic primary inguinal hernia in adult men (strength of recommendation IA). In a departure from this firm opinion presented by the EHS, the Shouldice method has been acknowledged to be acceptable as well [3]. Schumpelick emphasized the effectiveness of the Shouldice technique during his presentation at the 2011 EHS Congress in Ghent. Some questions can be asked considering these facts: Is the Shouldice technique the only nonmesh method that ensures good clinical results? Are any other tissue-based techniques effective in inguinal hernia repair if performed correctly?

The synthetic prostheses most often used in the inguinal area can create new clinical problems, such as foreign body sensation in the groin, discomfort, and abdominal wall stiffness, which may affect the everyday functioning of the patient [4]. Surgical-site infections, often with clinical symptoms delayed for many years, are more frequent after hernia treatment using mesh [5, 6]. Migration of the mesh from the primary site of implantation in the abdominal cavity is one of the most dangerous complications [7–9]. Intense chronic inflammatory process typically associated with foreign body reactions around the mesh prosthesis may produce meshoma or plugoma tumors, the treatment of which becomes a new surgical challenge [10–12]. Additionally, procreation and sexual function are reportly seriously affected after surgical hernia treatment with mesh [8, 13]. Thus, we are still far from accomplishing everything in the hernia surgical field, and complications remain the major clinical problem.

The observed complication rates and postoperative dysfunction have influenced many investigators to look for new hernia repair techniques or to modify old ones. An example of such efforts is the Desarda method, which was presented in 2001 and became a new surgical option for tissue-based groin hernia repair [14, 15]. Because the results of our prospective study involving the technique were promising, as were the results presented by other authors [16, 17], we performed a multicenter randomized double-blind clinical trial to compare the standard mesh-based Lichtenstein technique with the tissue-based Desarda technique.

## Materials and methods

### Patients

The patients were recruited in Poland from two clinical departments (the Department of General, and Endocrine Surgery and the Department of General, Gastrointestinal, and Cancer Surgery, Collegium Medicum in Bydgoszcz, Nicolaus Copernicus University of Torun) and one community surgical ward (General Surgery Ward, Community Hospital, Lodz). The local research and ethics committee approved the study protocol.

A total of 208 adult male Caucasian patients with primary inguinal hernias were randomly allocated intraoperatively to undergo one of the two repairs: Desarda tissue-based repair (D) or the classic Lichtenstein mesh repair (L). Patients with bilateral hernias were also included, but only one side was operated on. The final inclusion criterion was the assessment of the condition of the external oblique aponeurosis, with exclusion of patients with an aponeurosis that was divided, tiny, and/or weak. Patients with recurrent or strangulated hernias or mental disorders, those participating in other clinical trials, and those assessed on the American Society of Anesthesiologists (ASA) scale at >3 were also excluded from the study. Other exclusion criteria included a history of a forced hernia reduction with subsequent hospitalization, a history of infection, or the presence of any scar in the inguinal area.

**Fig. 1:**
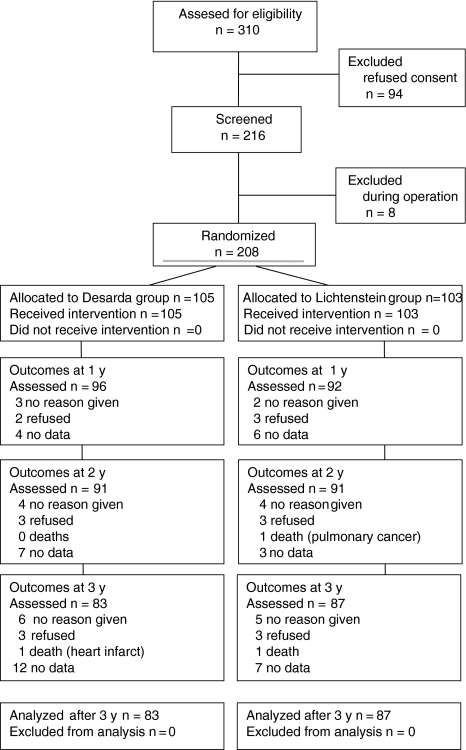
Trial flow chart

The participants were given detailed information on the trial and surgery. They each agreed to not be informed about the technique used until 2 years following the date of surgery, and each participant signed an informed consent form. The protocol details were discussed with the study team, and the surgical procedures were practiced to achieve standardization. Enrollment of eligible patients began on January 2005 and took place until June 2006. Patients were followed for a minimum of 3 years. The trial ended on June 30, 2009. More than 30% of eligible patients declined to give consent to be randomized (Fig. 1). Finally, 208 patients were blindly and randomly allocated to undergo one of the two open hernia repairs: Desarda or Lichtenstein procedure. The patient characteristics recorded were age, co-morbidities (Charlson Co-morbidity Index, ASA), and employment status (Table 1).

**Table 1:** Baseline health status characteristics, by operative method

Characteristic	Desarda (*n* = 105)	Lichtenstein (*n* = 103)	p*
Age (years): mean (SD)	50.2 (17.5)	54.1 (15.3)	0.094**
CCI: median, range	1 (0–4)	2 (0–3)	0.405***
ASA score: median (range)	1 (1–3)	2 (1–3)	0.484***
Co-morbidities (no.)			
Hypertension	12 (11.4%)	20 (19.4%)	0.127
Heart infarct	2 (1.9%)	4 (3.8%)	0.443****
Ischemic heart disease	9 (8.6%)	12 (11.6%)	0.498
Diabetes	6 (5.7%)	8 (7.8%)	0.592
Cerebral circulation insufficiency	8 (7.6%)	11 (10.7%)	0.479
Hepatitis C	1 (0.9%)	2 (1.9%)	1.000****
COPD	2 (1.9%)	0	0.498****
Peptic ulcer	4 (3.8%)	2 (1.9%)	0.683****
Chronic renal disease	0	2 (1.9%)	0.244****
BMI > 30 kg/m^2^	4 (3.8%)	2 (1.9%)	0.683****
Smoking	28 (26.6%)	23 (22.3%)	0.521****
Employment (no.)			
Student	7 (7.6%)	9 (8.7%)	0.412
Nonphysical	34 (32.4%)	31 (30.1%)	
Light physical	27 (25.7%)	33 (32.0%)	
Heavy physical	2 (1.9%)	4 (3.9%)	
None or retired	35 (33.3%)	26 (25.2%)	

*CCI* Charlson Co-morbidity Index, *ASA* American Society of Anesthesiologists, *COPD* chronic obstructive pulmonary disease, *BMI* body mass index

* *χ* ^2^ test, except: ** Student’s *t* test; *** Mann–Whitney *U* test; **** Fisher’s exact test

### Treatment

Using a standard protocol, all patients were given sedative premedication (7.5 mg midazolam) and one shot of antimicrobial prophylaxis (1.0 g cephazoline IV 30 min before surgery). In accordance with the patient’s preference or the anesthetist’s opinion, all operations were carried out under local (20.9 vs. 18.4% in D and L groups, respectively), regional (64.8 vs. 63.1%), or general (14.3 vs. 18.4%) anesthesia. The operations were performed by staff surgeons and surgeons in training, with equal proportions in both groups.

Randomization was achieved using computer-generated allotments that were disclosed to the surgeon via sealed envelope. Stratified randomization was used to ensure that an equal proportion of junior and senior surgeons performed the operations in both groups. The type of anesthesia was monitored during the study, but no intervention was necessary to ensure equal proportions in groups. The envelope was not opened until after the dissection and assessment of the external oblique aponeurosis had been performed because the condition of the aponeurosis was the final inclusion criterion.

**Fig. 2:**
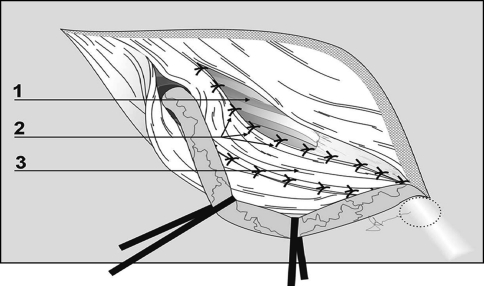
Desarda’s method. The undetached aponeurotic strip (*3*) is created and displaced from the anterior to the posterior wall of the inguinal canal. It was then secured to the abdominal internal oblique muscle (*1*) with interrupted sutures (*2*) and to the inguinal ligament

The Lichtenstein tension-free mesh repair was performed as described by Amid [18]. An 8 × 12 cm polypropylene mesh (Prolene; Ethicon, Somerville, NJ, USA) was trimmed to a foot-like shape to fit the inguinal floor. The mesh was sutured to the ligament of Poupart using a nonabsorbable continuous 2/0 suture (Prolene; Ethicon) and secured cranially using an absorbable 2/0 suture (Maxon; Covidien, Mansfield, MA, USA). The Desarda repair was performed as it was originally described in 2001 [14, 15] and presented in Fig. 2. Continuous nonabsorbable suture (2/0 Prolene; Ethicon) was used to secure the aponeurotic strip to the inguinal ligament laterally, and the strip was secured to the internal oblique muscle medially with interrupted, absorbable sutures (2/0 Maxon; Covidien). Particular attention was paid to identify and preserve the nerves of the inguinal area; whenever this was not possible, the nerves were excised. All intraoperative variables were recorded and compared. After the inguinal canal had been opened, the hernias were described using the Gilbert-modified Robbins–Rutkow classification system as follows: type 1, indirect hernia with normal internal ring; type 2, indirect hernia with internal ring enlarged but <4 cm; type 3, indirect hernia with internal ring enlarged >4 cm; type 4, direct hernia with destroyed posterior wall of the inguinal canal; type 5, direct hernia with defect next to the pubic tubercle; type 6, pantaloon hernia; type 7, femoral hernia. For both techniques, the skin was closed with continuous nonabsorbable suture. Patients were encouraged to resume normal activities as soon as possible.

### Follow-up

Inpatients were examined by a blinded investigator until discharge and seen during follow-up appointments at 7, 30 days, and 6, 12, 24, and 36 months after surgery. The appointments on day 7 were performed during the patients’ visits to outpatient surgical departments; and the follow-up appointments after day 7 were performed in the departments’ examination rooms. Both the patients and controlling investigators were blinded to the hernia surgery method used. The investigator who was performing the follow-up physical examinations and patient assessments was a surgeon in each department who did not perform the surgeries in this study. The data were collected in computer protocols of Sharepoint Portal Server System (Microsoft, Edmond, WA, USA) after it was adapted to perform in clinical trials by the authors.

Recurrences and other complications were recorded. Pain was measured using a visual analog scale (VAS), which ranged from 0 (no pain) to 100 (maximum, unbearable pain). Additionally, pain was recorded with the use of the Sheffield scale: 0, no pain; 1, no pain at rest but it appears during movement; 2, temporary pain at rest and moderate during movement; 3, constant pain at rest and severe during movements. Return to normal activity was described as the patient’s ability to perform elementary activities [i.e., dressing, walking, bathing (basic activity)]; usual activities at home [i.e., preparing food, cleaning house (home activity)]; and returning to all previously performed activities (work activity).

### Outcomes

The aim of the present study was to test the hypothesis that the Desarda repair is as effective as the standard Lichtenstein procedure, allowing successful hernia repair without mesh. The primary outcomes were hernia recurrence and chronic pain, defined as moderate (VAS 30–54) or strong (VAS > 54) pain lasting more than 6 months after surgery. The secondary outcomes were general and local complications, length of time to return to various levels of everyday activity, foreign body sensation, and abdominal wall stiffness in the groin area.

### Statistical analysis

The study was designed to detect a 15% difference in recurrence rate between the groups, with a sample of at least 72 hernias per group, a power of 0.8, and an α error of 0.05. The estimated loss of participants available for assessment during the 3-year follow up was 30%; therefore, a group of at least 104 patients was planned to be enrolled for each group. Patients who were lost during follow-up were excluded from the analysis; only the patients who completed the study were included.

Student’s *t* test and Mann–Whitney *U* test were used for analysis of quantitative data. The normality of distribution was checked with the KS test. Pearson’s *χ* ^2^ and Fisher’s exact tests were used for analysis of qualitative data. Differences were considered statistically significant at *p* < 0.05. SPSS version 17.0 (SPSS, Chicago, IL, USA) and Statistica.PL version 8.0 (StatSoft, Krakow, Poland) software programs were used for statistical calculations.

## Results

There were a total of 105 patients in the D group and 103 in the L group, comprising the two study arms. Baseline characteristics, including demographics, co-morbidities, and occupation, were similar in the two groups (Table 1). Hernia characteristics are given in Table 2. Intraoperative variables (i.e., nerve excision, lipoma, opening of the hernia sac, among others) were comparable, with no significant differences found (data not shown).

**Table 2:** Characteristics of operated hernias, by operative method

Characteristic	Desarda (*n* = 105)	Lichtenstein (*n* *=* 103)	p*
Bilateral hernia (no.)	4 (3.8%)	8 (7.8%)	0.888**
Right side operation (no.)	61 (58.1%)	58 (56.3%)	0.866***
Left side operation (no.)	44 (41.9%)	45 (43.7%)	0.795***
Duration of hernia (months)	7 (1–108)	12 (1–240)	0.337
Nonreducible hernia (no.)	6 (5.7%)	5 (4.8%)	1.000**
Local preop. VAS pain score	33 (0–91)	34 (0–86)	0.255
Local preop. Sheffield pain score	1 (0–3)	1 (0–3)	0.810
Size of hernia orifice (cm)	3 (1–9)	3 (1–8)	0.586
Hernia type by Robins–Rutkow classification (no.)	2 (1–6)	2 (1–6)	0.207
Median and range of types			
Type			
1	17 (16.2%)	21 (20.4%)	
2	45 (42.8%)	51 (49.5%)	
3	17 (16.2%)	11 (10.7%)	
4	20 (19%)	15 (14.6%)	
5	1 (0.9%)	2 (1.9%)	
6	5 (4.8%)	3 (2.9%)	

Results are the median and range unless otherwise stated

*VAS* visual analog scale

* Mann–Whitney *U* test, except: ** Fisher’s exact test; *** *χ* ^2^ test

Of the 208 patients operated on, all were examined at the 7-day, 30-day, and 6-month follow-up visits. Afterward, 188 (90.4%) came for a physical examination and questioning at 1 year, 182 (87.5%) at 2 years, and 170 (81.7%) at 3 years. The response rate was similar in the two groups. A detailed trial flow chart is presented on Fig. 1.

There were two (1.9%) recurrences in each study group during the 3-year time period (*p* = 1.000). In the D group, one recurrence was found above the re-created deep inguinal ring in the triangle between the inguinal ligament, the strip of external oblique aponeurosis, and the spermatic cord. The second recurrence in the D group was found as a weakening of the posterior wall of the inguinal canal. The recurrences in the L group were found in their typical localization, close to the pubic tubercle. No early recurrence (<1 year) was seen.

There was no significant difference between the D and L groups in regard to pain reported after 6 months via the VAS score (mean 7.9 vs. 7.7 mm, respectively; *p* = 0.877) and in the Sheffield scale (D vs. L: mean 0.6 vs. 0.5, respectively; *p* = 0.372). After the VAS results were transformed to a descriptive pain scale, no differences were noted there either. Patients from the D and L groups reported mild pain (VAS 1–29): 49 (46.6%) and 46 (44.6%) patients, respectively (*p* = 0.464). Five (4.8%) and three (2.9%) patients from the D and L groups, respectively (*p* = 0.464) reported chronic pain; it was classified as moderate pain (VAS 30–55). No strong pain (VAS > 55) was observed after 6 months of follow-up.

The rates of early and late complications were similar in the two groups (Table 3). The number of seromas was comparable for the D and L groups 7 days after the surgery, but the number was higher in the L group at the 30-day follow-up (0/105 vs. 8/103, respectively; *p* = 0.004).

**Table 3:** Outcomes and postoperative complications, by operative method

Parameter	Desarda (*n* = 105)	Lichtenstein (*n* = 103)	p*
Testicular edema (no.)			
7 Days	8 (7.7%)	10 (9.7%)	0.607
30 Days	6 (5.9%)	6 (5.8%)	0.972
6 Months	0	0	
Testicular atrophy (no.)	0	0	
Inguinal hematoma (no.)	8 (7.7%)	7 (6.8%)	0.789
Hematomas needing drainage (no.)	1 (0.9%)	2 (1.9%)	0.621**
Ecchymosis (no.)	5 (4.8%)	5 (4.8%)	1.000**
Seroma (no.)			
7 Days	4 (3.8)%	6 (5.8%)	0.508**
30 Days	0	8 (7.8%)	0.004**
Surgical-site infection (no.)	1 (0.9%)	2 (1.9%)	1.000**
Return to basic activity (days)	1 (1–7)	1 (1–7)	0.221***
Return to home activity (days)	7 (2–21)	7 (2–30)	0.224***
Return to work activity (days)	21 (7–90)	20 (4–90)	0.210***

Results are the median and range unless otherwise stated

* *χ* ^2^ test, except: ** Fisher’s exact test; *** Mann–Whitney *U* test

Return to basic and home activities was achieved after comparable mean times in the two groups. Although return to work activity occurred later in the D group, the difference was not significant at any of the time points (Table 3). The percentage of patients with foreign body sensation, abdominal wall stiffness, and subjective loss or change in sensation in the operated groin was higher in the L group than in the D group, but the difference never reached statistical significance (Table 4).

**Table 4:** Patients’ subjective assessment of the operated area at the 12-, 24-, and 36-month follow-ups

Parameter	Desarda (*n* = 96)	Lichtenstein (*n* = 92)	p*
12-Month follow-up^a^			
Foreign body sensation	13 (14.6%)	17 (18.1%)	0.525
Abdominal wall stiffness	14 (15.7%)	20 (21.3%)	0.335
Loss or change of sensation in the operated groin	36 (40.4%)	42 (44.7%)	0.563
24-Month follow-up^b^			
Foreign body sensation	14 (15.2%)	16 (17.6%)	0.666
Abdominal wall stiffness	15 (16.3%)	18 (19.8%)	0.541
Loss or change of sensation in the operated groin	38 (41.3%)	41 (45.1%)	0.609
36-Month follow-up^c^			
Foreign body sensation	10 (12.2%)	16 (18.8%)	0.238
Abdominal wall stiffness	10 (12.2%)	19 (22.3%)	0.083
Loss or change of sensation in the operated groin	36 (40.4%)	40 (38.8%)	0.386

Data are expressed as the number of patients

* *χ* ^2^ test

^a^There were 96 patients in the Desarda group and 92 in the Lichtenstein group

^b^There were 91 patients in the Desarda group and 91 in the Lichtenstein group

^c^There were 83 patients in the Desarda group and 87 in the Lichtenstein group

## Discussion

No significant differences in clinical outcomes were observed during a 3-year follow-up of adult male patients with a primary inguinal hernia operated on with either the Desarda or the Lichtenstein technique. Excluding seroma formation, the frequency of complications was similar for the two groups.

Currently, the results of hernia treatment, even those that have taken into account the EHS guidelines, vary from moderate to excellent. The mean recurrence rate for the standard Lichtenstein procedure is about 1% in hernia-specialized centers but can be much higher in community hospitals (about 4%), and the reported rate even reaches 18% in some articles [19]. The data published so far for other mesh techniques vary: 0 to 4.2% recurrences for Prolene Hernia System (PHS) [20], 0 to 4% for Rutkow [21], 1.6 to 19.0% for the Transabdominal Pre-Peritoneal inguinal hernia repair (TAPP) [19]. The summarized frequency of postoperative complications reported in the available literature is between 15 and 28% [22, 23]. When active postoperative monitoring is applied, the frequency can even reach 50% [19]. The most frequently reported complications were hematoma, seroma, surgical-site infection, chronic pain, and recurrence [24]. Death and major worsening of the treated patients’ quality of life were rare but also reported [24, 25]. These data suggest the need for further investigation of the clinical problem.

An intense global effort to improve the results of inguinal hernia treatment is ongoing. Commercially available lightweight polypropylene meshes, composed meshes, and many biologic prostheses are being tested. The scientific work of optimizing hernia surgery and lowering the number of complications is still in progress. We are of the opinion that tissue-based techniques are not out of the realm of consideration in this field.

The Desarda technique for inguinal hernia repair is a new tissue-based method. Despite the objections presented by some authors [26, 27], application of the external oblique muscle aponeurosis in the form of an undetached strip (which makes the posterior wall of the inguinal canal stronger) has been established as a new concept in tissue-based hernia repair. The technique is original, new, and different from the historical methods using the external oblique aponeurosis, proposed initially by McArthur [28] and Andrews or Zimmermann [29].

In our opinion, this newly proposed repair method satisfies the principles of “no tension” presented by Lichtenstein. The aponeurotic strip is displaced from the anterior to the posterior wall of the inguinal canal without additional tension at the posterior wall. The concept of an undetached, movable aponeurotic strip that “physiologically” enforces the posterior wall of the inguinal canal is original and interesting [30, 31]. When considering the Desarda technique as “dynamic enforcement” of the inguinal canal’s posterior wall, the Lichtenstein method can be called “prosthetic enforcement.” The author of the first method hypothesizes that a naturally displaced and movable aponeurotic strip is far more “physiological” than the scar tissue produced around a synthetic prosthesis for creating a mechanism against reherniation.

What can be postulated against this technique is that the originally unhealthy tissue is used for the repair. There is evidence supporting the role of matrix metalloproteinases (MMPs) and their inhibitors (tissue inhibitor of MMPs, or TIMPs) in abdominal wall connective tissue degeneration leading to hernia formation. The coincidence of hernia and aortal aneurysm and other diseases in which the etiopathology originates from connective tissue is well known. Hernia formation and recurrence is associated with altered collagen metabolism manifested by a decreased type I:III collagen ratio. The Shouldice technique, which is still recommended and accepted worldwide, is tissue-based as well. To date, there has been no comparison study on the aponeurotic tissue and the transversalis fascia. The properties of inguinal connective tissue are being generalized mainly from studies on the transversalis fascia. It should be noted that the genetic and biochemical changes are found in only 20 to 30% of patients with hernias. Assuming that there are about 15 to 20% of recurrences after some tissue-based techniques, 80% of patients survive without recurrence for the remainder of their lives. It might be postulated that there is a population of hernia patients—actually, most of the patients—in whom tissue-based techniques could be used safely. The future challenge in herniology is finding a method to identify this population before surgery.

In our study, there were no statistically significant differences between the patients enrolled and randomized to the Desarda and Lichtenstein groups. The recurrence rate was the same in both groups. In one case in the Desarda group, the recurrence was obviously the result of a technical error. The aponeurotic strip created was too long, resulting in a large newly formed deep inguinal ring and reherniation. In the second case of recurrence, weakening of the entire posterior wall was found during reoperation, but no typical reherniation was seen. In the Lichtenstein group, the recurrences were typical. This additionally supports the idea that surgical technique is crucial for a good final result.

Although chronic pain has been defined as lasting >3 months by the International Association for the Study of Pain [32], we defined chronic pain as pain lasting ≥6 months due to the use of synthetic materials for the hernia repair and taking into account the fact that the inflammatory response to foreign material may last longer. This approach has been used by many other authors [33, 34] and is recommended in the latest publications [35]. At the early postoperative time points (7 and 30 days), the pain was slightly higher in the Desarda group; but the difference never reached significance. After the VAS scale was transferred to a descriptive scale (Verbal Rating Scale, VRS) no differences at any the follow-up time points, including at 6 months, were observed. We excluded data from the early postoperative days because of the many protocol deviations observed, different anaesthesia applied, and multiple medications taken by the participants. In another recent publication of our study results on early postoperative pain after the Desarda and Lichtenstein operations, no significant differences were found [36].

The percentage of other early and late complications was comparable. The higher ratio of seromas after use of the Lichtenstein method can be explained by the influence of the synthetic mesh on surrounding tissues. This is consistent with other studies and the known influence of polypropylene on tissue [37, 38]. Foreign body sensation and abdominal wall stiffness were expressed by 12 to 16% of the Desarda group patients and 17 to 22% of the Lichtenstein group patients at different time points, and the results are within the range (4.5–43.8%) reported by other authors for mesh techniques [39, 40]. Surprisingly, these mesh-related sensations were experienced similarly by patients from both groups and did not change even after the participants were informed of the technique used after 2 years of follow-up.

To the best of our knowledge, this is the first report of a randomized clinical trial comparing the Desarda and Lichtenstein techniques. Previously, Mitura and Romanczuk have published the results of a 6-month follow-up study of the Desarda and Lichtenstein approaches [16]. They observed no recurrence, and pain after 6 months was comparable in the two groups (VAS scores were 8 vs. 11 in the Desarda and Lichtenstein groups, respectively; *p* = 0.691). Situma et al. [41] presented their short-term results of Desarda versus modified Bassini inguinal hernia repair, concluding that there was no difference between these two techniques in regard to pain and return to normal activity. Other results, published by Desarda and his group, were based on a comparison of his technique and the Lichtenstein technique [42]. They reported no recurrence among the 269 Desarda group patients and 1.97% recurrence among the 225 mesh group patients; 6.49% of patients from the mesh group and no patients in the Desarda group reported chronic pain at 1 year after surgery. In our opinion, despite some methodologic inadequacies in the presented articles, the Desarda method merits more attention and further investigation by other authors.

Paradoxically, in the modern world the cost of the medical treatment becomes the real issue. The cost of inguinal hernia treatment, a tiny fraction of all health expenses, is not insignificant, however, especially in developing countries in Asia or Africa. One indisputable advantage of Desarda technique is its low cost. That is why many published articles recently demonstrated an interest in the technique [41, 43, 44]. The cost of the Desarda operation is low because a synthetic prosthesis is not needed. The price of composite meshes or even heavy polypropylene meshes, as well as their accessibility, could be important issues in developing countries. We confirmed that even the inguinoscrotal hernias (Rutkow types 3, 4, and 6), which are frequently seen in African and Asian countries, can be successfully treated with the Desarda technique.

Economic issues are not the only considerations. The use of synthetic material is still controversial in young patients. The effect of polyproplylene placement or other synthetic mesh inside human organism for a lifetime is still unknown. Also, data are appearing about sexual impairment after mesh implantation; and as a result, many surgeons try to avoid mesh prostheses for hernia treatment in young patients. Also, the Desarda method, a tissue-based technique, can be used in a contaminated surgical field, usually seen during operations for strangulated hernias.

## Conclusions

Our random controlled trial confirmed that the results of inguinal hernia treatment with the Desarda technique are similar to the results after standard Lichtenstein operations over a 3-year time period. Based on these results, the technique has the potential to enlarge the number of tissue-based methods available to treat groin hernias. The most evident indications for use of the Desarda technique include use in young patients, in contaminated surgical fields, in the presence of financial constraints, or if a patient disagrees with the use of mesh.
